# Screening of *CYP1B1* and *MYOC* in Moroccan families with primary congenital glaucoma: Three novel mutations in *CYP1B1*

**Published:** 2010-07-02

**Authors:** Latifa Hilal, Soraya Boutayeb, Aziza Serrou, Loubna Refass-Buret, Hafsa Shisseh, Fatiha Bencherifa, Mohammed El Mzibri, Bouchra Benazzouz, Amina Berraho

**Affiliations:** 1 Laboratoire de Génétique et de Physiologie Neuroendocrinienne, Equipe des Bases Moléculaires de Maladies Génétiques, Université Ibn Tofaïl, Faculté des Sciences, Kénitra, Morocco; 2 Service d’Ophtalmologie B, Hôpital des Spécialités, CHU Ibn Sina, Equipe de Recherche sur les maladies oculaires, Faculté des Médecine et de Pharmacie, Rabat, Morocco; 3 CNESTEN BP1382 RP10001, Rabat, Morocco

## Abstract

**Purpose:**

To investigate the contribution of cytochrome P4501B1 (*CYP1B1*) and myocillin (*MYOC*) mutations to primary congenital glaucoma (PCG) in Moroccan families.

**Methods:**

This study included 90 unrelated families with PCG and 100 normal control individuals. Two previously reported *CYP1B1* mutations (g.4339delG and p.G61E) were first screened by polymerase chain reaction-restriction fragment length polymorphism (PCR-RFLP). The coding exons of *CYP1B1* were sequenced in g.4339delG- and p.G61E-negative or heterozygous probands. Then the coding exons of *MYOC* were sequenced in patients who had no mutation in *CYP1B1* or carried heterozygous *CYP1B1* mutation.

**Results:**

Twelve *CYP1B1* mutations were identified in 43 PCG pedigrees. Three of them were novel (p.R163C, p.C470Y, and g.4330–4331delTG) and associated with moderate to severe phenotypes. Two novel intronic polymorphisms in *CYP1B1* were identified in addition to those previously described. The g.4339delG was the most frequent mutation detected in 31 families (34.44%), followed by the p.G61E in seven families (7.77%). The remaining mutations (p.R163C, p.E173K, g.4330–4331delTG, p.E229K, p.R390S, p.R368H, p.R469W, p.C470Y, and g.7901–7913del13bp) were infrequent. One family with the p.R390S mutation showed both PCG and primary open angle glaucoma (POAG) phenotypes. One proband was heterozygous for p.T193K mutation in *MYOC*. This mutation has been initially associated with POAG, but never with PCG.

**Conclusions:**

Our results support that mutations in *CYP1B1* are a major cause for PCG in the Moroccan population with a predominance of the g.4339delG mutation. Furthermore, these results demonstrate the diversity of *CYP1B1* mutations, while suggesting a modest role of *MYOC* in Moroccan PCG.

## Introduction

Primary congenital glaucoma (PCG, OMIM 231300) is characterized by a marked increase of intraocular pressure at birth or early childhood, large ocular globes (buphthalmos), corneal edema, and Haab’s striae [1-3]. It is associated with developmental defects in the anterior chamber and is mainly inherited as an autosomal recessive disorder with incomplete penetrance [4,5]. The incidence of PCG varies among geographic locations and ethnic communities, from 1:10,000–20,000 in western countries, to 1:2,500 and 1:250 in inbred Slovakian Gypsy and Saudi Arabian populations, respectively [3]. So far, three genetic loci have been linked to PCG: GLC3A (chromosome 2p21), GLC3B (chromosome 1p36), and GLC3C (chromosome 14q24.3). The only gene that has been identified is the cytochrome P4501B1 gene (*CYP1B1*) linked to GLC3A [4,6].

Mutations in *CYP1B1* are the major cause of PCG with more than a hundred that have been reported in the Human Gene Mutation Database [2]. Nonetheless, limited role of myocillin (*MYOC* at chromosome 1q24–25) [7-9], forkhead box C1 (*FOXC1* at chromosome 6p25) [10], and latent transforming growth factor beta binding protein 2 (*LTBP2*; close to the GLC3C locus) [11,12], has been suggested in PCG pathogenesis. In some studies, *CYP1B1* mutations have been reported to be associated with primary open-angle glaucoma (POAG, OMIM 137760) [13-17]. However, their contribution to the occurrence of POAG was controversial. Recently, extended studies and functional analysis demonstrated that heterozygous *CYP1B1* mutations with absent or reduced function can be considered as a risk factor for POAG [18,19]. *CYP1B1* mutations are also detected in Rieger’s anomaly [2,20,21], Peters’ anomaly, and Sturge-Weber syndrome [22]. In certain pedigrees, both PCG and POAG segregate [23-25]. Some patients with PCG exhibit mutations in both *CYP1B1* and *MYOC* [7,26]. The human *CYP1B1* gene consists of three exons, two of which are coding. The CYP1B1 protein is a member of the cytochrome P450 superfamily (subfamily I) [27]. The only reported mutational analysis of *CYP1B1* in PCG patients from Morocco has been performed on isolated cases and identified only two mutations: g.4339delG and p.G61E [28].

To further evaluate the role of *CYP1B1* mutations in Moroccan PCG, as well as the presumptive contribution of *MYOC* in the disease, we investigated the mutation spectrum of these two genes in a large cohort of unrelated Moroccan PCG families.

## Methods

### Patients

This research adhered to the tenets of the Declaration of Helsinki. The patients were referred for evaluation of their glaucoma to the Department of Ophthalmology of the University Ibn Sina Hospital in Rabat, Morocco. A total of 187 individuals were included in this study. They belonged to 90 unrelated Moroccan PCG families residing in different regions of Morocco and comprising 90 probands, 18 affected and 79 non-affected relatives. Control DNAs (n=100) were obtained from randomly selected healthy adults. All subjects gave their informed consent. All patients were examined by at least one ophthalmologist.

Ophthalmological examinations included biomicroscopy, gonioscopy, and measurement of intraocular pressure (IOP) by Perkins tonometers, and optic nerve examination. IOP and corneal diameter were measured under general anesthesia. Inclusion criteria were: increased corneal diameter (>12.0 mm), raised IOP (>21 mHg) with or without Haab’s striae, and optic disc changes whenever anterior segment conditions made fundus examination possible. Additional inclusion factors were epiphora and photophobia. Trabeculectomy was the initial surgical procedure. Trabeculectomy with Mitomycin C was performed on second attempt. Individuals presenting with other ocular or systemic anomalies were excluded. Clinical features of 20 of the 90 probands with mutations are shown in Table 1.

**Table 1:** Clinical features of probands with primary congenital glaucoma.

**Patient ID**	**Mutation**	**Gender**	**Csg. parent C/NC**	**Age at onset**	**Age at diag.**	**Laterality**	**IOP Max (mmHG) (R/L)**	**Corneal Diam. (mm; R/L)**	**C/D ratio (mm;R/L)**	**Corneal opacities R/L**	**Corneal Edema R/L**	**Haab’s striae R/L**	**Trab R/L**	**LVA R-L**	**Prognosis**
**A- Patients with *CYP1B1* mutation**															
PCG-1-II.5	p.E173K/ g.4339delG	M	C	2m	2m	B	45/50	14/14	#/0.8	+/+	+/+	+/+	Mult/Mult	#-#	NRSM
PCG-11-II.1	g.4339delG/ p.V364M	M	NC	2m	17m	B	24/38	13/14	0.1/#	+/+	+/+	#/#	1/1	#-#	bad
PCG-14-II.2	p.R368H/w	M	NC	2m	9m	B	26/47	11.5/16	0.1/#	-/+	−/−	-/+	1/1	#-blind	NRSM
PCG-20-II.2	p.G61E/p.G61E	M	NC	5y	6y	B	29/28	14/15	0.8/0.9	+/+	−/−	+/+	1/1	#-#	bad
PCG-28-II.2	p.E173K/ g.4339delG	F	C	birth	37d	B	34/30	14/13.5	0.6/0.6	+/−	+/+	−/−	1/2	blind-3/10	#/#
PCG-29-III.3	g.4339delG/p.G61E	M	NC		8m	B	30/25	15/15	#/0.7	+/+	−/−	+/+	1/1	#-#	NRSM
PCG40-IV.6	p.R390S/ p.R390S	M	C	birth	16d	B	>50/>50	12/11.5	#/#	+/+	+/+	+/+	Mult/Mult	-enucl	bad
PCG-45-II.3	p.G61E/p.G61E	F	C		14y	B	23/38	15/15	0.3/0.7	-/+	+/+	-/+	1/1	1/10-blind	bad
PCG-47-II.3	p.G61E/p.G61E	M	NC	2m	3m	B	32/35	15/14	0.6/0.5	+/+	-/+	+/+	2/2	#-#	bad
PCG-58-V.2	g.7901-7913del	F	C	4m	9m	B	35/44	14/14	#/#	+/+	+/+	+/+	1/1	LP-LP	bad
PCG-64-III.1	g.4330–4431delTG/ g.4339delG	F	NC	birth	45d	B	28.5/32.6	16/16	0.5/0.7	+/+	+/+	+/+	1/Mult	2/10–2/10	NRSM
PCG-75-IV.1	p.R469W/ p. R469W	F	C	6m	9m	B	/26	14/13	0.4/0.3	−/−	−/−	−/−			
PCG-79-III.3	g.4339delG/p.G61E	M	NC	3m	4m	B	28/38		1/1	+/+	+/+	+/+	1/1	#-#	bad
PCG-84-V.2	g.4330–4431delTG/g.4330–4431delTG	F	C	birth	3m	B	27/31	11/12	#/#	-/+	−/−	−/−	1/		
PCG-89-V.3	p.G61E/p.G61E	F	C	birth	2m	B	28/28	14/12	0.6/0.3	+/+	+/+	+/+	1/1	1/10–1/10	NRSM
PCG-95-III.1	g.4339delG/g.7901-7913del	F	NC	1m	5m	B	53/49	14/14	#/#	+/+	+/+	+/+	1/1	#/#	bad
PCG-97-II.3	p.G61E/p.G61E	M	C	birth	11y	B	16/22		0.3/1	-/+	-/+	-/+	1/2	2/10–1/10	bad
PCG-100-II.3	p.C470Y/ p.C470Y	F	C	birth	50d	B	42.2/44.7	12–13/11	#/#	+/+	+/+	+/+	1/1	#/#	bad
PCG-101-III.3	p.R163C/w	F	C	3m	7m	B	20/21	13/14	#/#	-/+	+/+	−/−	/1	LP/LP	
**B-Patient with *MYOC* mutation**															
PCG-009-II.3	p.T193K	M	NC	2m	6m	B	32/36	15/15	#/#	+/+	+/+	+/+	2/2	#/#	NRSM

A- Probands with *CYP1B1* mutations. Clinical features of probands with homozygous g.4339delG, which are not shown in this table, are reported in Berraho et *al*. (article in preparation), B- Proband with *MYOC* mutations. Unknown phenotypic features are left in blank, Age at onset: Age of symptoms apparition, Age at diagnosis: age at the first examination where the PCG was diagnosed, M: male, F: female, d: days, m: month, y: year, Csg: Consanguinity, C: consanguineous, NC: non consanguineous, IOP: intraocular pression, C/D: cup-to-disk ratio, Trab: trabeculectomy, Mult: multiple, enucl: enucleated, LVA: Last visual acuity, R: right eye, L: left eye, LP: light perception, # unable to measure, NRSM: no response to surgery and medication.

### *CYP1B1* and *MYOC* mutation screening

Patient's genomic DNA was extracted from peripheral blood leucocytes using standard phenol/chlorophorm procedure. Polymerase chain reaction–restriction fragment length polymorphism (PCR-RFLP) was performed for rapid detection of g.4339delG and p.G61E mutations. The coding exons of *CYP1B1* were then analyzed by direct sequencing in g.4339delG- and p.G61E-negative or heterozygous cases. Then the coding exons of *MYOC* were sequenced in patients who had no mutation in *CYP1B1* or carried heterozygous *CYP1B1* mutation. When a mutation was found, parents and all available related family members were tested.

The g.4339delG mutation abolishes a Hae II restriction site and the p.G61E creates a TaqI site. Genomic DNA of all patients was first amplified by PCR using primers surrounding the two mutations. The PCR-products were then digested by Hae II and TaqI according to the conditions specified by the manufacturers (Promega, Madison, WI), and separated on 2% agarose gel electrophoresis. Exons 2 and 3 of *CYP1B1* as well as exons 1, 2, and 3 of *MYOC* and their flanking intronic regions were amplified by PCR using intronic primers close to the intron/exon boundary. They were then sequenced using intronic and internal primers. PCR products were purified using the “PCR Clean-Up System Kit” (Promega) and sequenced on an ABI PRISM 3100 automated sequencer (Applied Biosystems, Foster City, CA). All the primers’ sequences used in this study are shown in Table 2.

**Table 2:** Primer sequences used in this study.

**Gene**	**Primer**	**Exon**	**Sequence (5’-3’)**	**Annealing temperature °C**	**Product size (bp)**
*CYP1B1*	C2F^1,2^	2	ACCCAACGGCACTCAGTC	58	1232
	C2R^1^		CCCTGCTTGCAAACTCAGC		
	C2.1F^2,3^		GCTCCTGTCTCTGCACC	58	636
	C2.1R^2,3^		GCCTCGGGTCGAGGAAG		
	C2.2F^2^		CTTCTTCACGCGCCAGC		651
	C2.2R^2^		CATATTCTGTCTCTACTCCGCC		
	C2.3F^2,4^		ATGGCTTTCGGCCACT	60	264
	C2.3R^2,4^		GGGGTCGTCGTGGCTGTAG		
	C3F^1,2^	3	AATGGGAAAGACAGCATTAGTC	60	1007
	C3R^1,2^		ATGAAGAACCGCTGGGTATG		
	C3.1F^2^		AGTGAGAAATTAGGAAGCTGTTTT		595
	C3.1R^2^		AGCCAGGATGGAGATGAAGA		
*MYOC*	M1F^1^	1	GCCACCTCTGTCTTCCCC	60	853
	M1R^1^		CTCTAGGAGAAAGGGCAGGC		
	M1.1F^2^		CAGGCACCTCTCAGCACAG		
	M1.1R^2^		AGCCCCTCCTGGGTCTC		406
	M1.2F^2^		ACCCAACGCTTAGACCTGG		432
	M1.2R^2^		TGTAGCAGGTCACTACGAGCC		
	M2F^1^	2	CCACATCCAGCTAATTCTTTTG	58	553
	M2R^1^		AGACCTGCTCTGACAAGGGA		
	M3F^1^	3	CAGACGATTTGTCTCCAGGG	55	1020
	M3R^1^		GAAAGCAGTCAAAGCTGCCT		
	M3.1F^2^		CATGATCATTGTCTGTGTTTG		521
	M3.1R^2^		GCTGTAAATGACCCAGAGGC		
	M3.2F^2^		GAGAAGGAAATCCCTGGAGC		500
	M3.2R^2^		CCAGGAGCCCTGAGCATC		

In the “primer” column, F indicates forward primer and R indicated reverse primer. ^1^Primers used for amplification of *CY1B1* and *MYOC* genes, ^2^primers used for DNA sequencing, ^3,4^primers used for p.G61E and g.4339delG screening, by PCR-RFLP, respectively.  bp: base pair.

### Computational analysis

Two homology-based programs PolyPhen (Polymorphism Phenotyping) [29,30] and SIFT (sorting intolerant from tolerant homology) were used to assess the functional effect of the substituted amino-acid [31,32]. Polyphen PSIC (position specific independent counts) scores of >2.0 indicate that the variation is probably damaging to protein function. Scores of 1.5–2.0 are possibly damaging, and scores <1.5 are likely benign. The SIFT threshold score of less than 0.05 was considered to be deleterious to the protein. We also used PolyPhen-2 program (PolyPhen v.2) whose performance is consistently superior to that of PolyPen [33]. Multiple alignments of CYP1B1 from different species were preformed using clustalW software.

### Mutation nomenclature

Mutations and polymorphisms were named based on the genomic DNA sequences U56438 and NT_004487 of *CYP1B1* and *MYOC*, respectively.

## Results

### Clinical features of patients

To investigate the role of *CYP1B1* mutations in Moroccan patients with PCG, we studied a total of 90 unrelated families affected by the disease. The presumed mode of inheritance of PCG according to pedigree analysis was autosomal recessive in all families except one who showed a pseudo-dominance mode (data not shown). Consanguinity was found in 47 of the 90 families (52%). More than one individual were affected in 27 families (30%). Among the 90 probands, 52 (58%) were male and 38 (42%) were female. The age of the onset of disease ranged from one day to 6 months (median 26 days).

At the time of the first specialized consultation (time of diagnosis), their ages ranged from 15 days to 19 months (median 7 months). The disease was bilateral in 82 patients (91.11%). Severe opacities were present in 75 eyes (41.66%), edema in 118 eyes (65.55%) and Haabs striaes in 38 eyes (21.11%). Corneal diameters, measured under general anesthesia, ranged from 11 to 18 mm with an average of 14.05±1.8 mm. The mean measured IOP before the first surgical procedure was 30.6±7.5 mmHg. The average cup-to-disk size ratio was 0.67±0.2. Trabeculectomy was performed as the initial surgical procedure in 126 eyes (70%), and surgical data were not available for 38 eyes (21.11%). Forty eyes (22%) needed more than one surgery. Two eyes were blind at the time of diagnosis. Ten others became blind during the follow-up, and finally one eye had to be enucleated due to the consequences of uncontrollable IOP.

### *CYP1B1* mutational analysis

**Figure 1:**
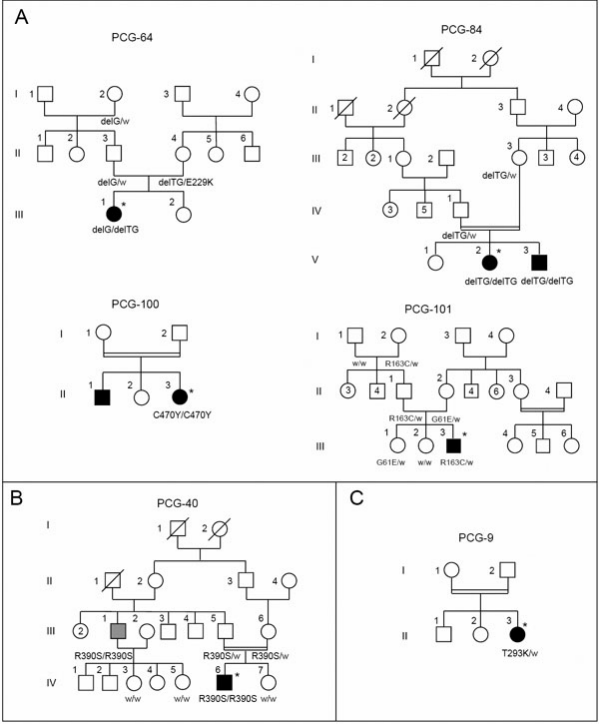
Pedigrees of PCG families with *CYP1B1* or *MYOC* mutations. **A**: Families with the *CYP1B1* novel mutation (p.R163C, p.C470Y, and g.4330–4331delTG) identified in this study. **B**: Family PCG-40 with the *CYP1B1* p.R390S mutation showing variable expression of the PCG phenotype. The proband (PCG-40-IV.6) was affected with PCG, while his uncle (PCG-40-III.1; gray symbol) developed POAG at the age of 45. **C**: Family PCG-9 with *MYOC* mutation. Deceased individuals are denoted by diagonal slashes, and consanguineous marriages by double lines. Asterisks indicate probands. Genotypes in available subjects are indicated below the symbols. delG: g.4339delG, delTG: g.4430–4431delTG, w: normal allele.

A total of 11 distinct *CYP1B1* mutations was identified in 43 (47.77%) of 90 unrelated probands: 8 substitutions (g.3987G/A, g.4292C/T, g.4322G>A, g.7927G/A, g.7940G>A, g.8005C>A, g.8242C/T, and g.8246C/A) predicting missense mutations (p.G61E, p.R163C, p.E173K, p.V364M, p.R368H, p.R390S, p.R469W, and p.C470Y) and 3 nucleotide deletions (g.4339delG, g.4430–4431delTG, and g.7901–7913delGAGTGCAGGCAGA [g.7901–7913del13bp]) resulting in three frameshift mutations followed by stop codons at position 179, 222, and 422, respectively (Table 1, Figure 1). For the second deletion, because of a three GT repeat at nucleotide position 4427–4432, it is not possible to determine exactly which of the positions is deleted. This deletion was arbitrarily named g.4430–4431delTG.

**Figure 2:**
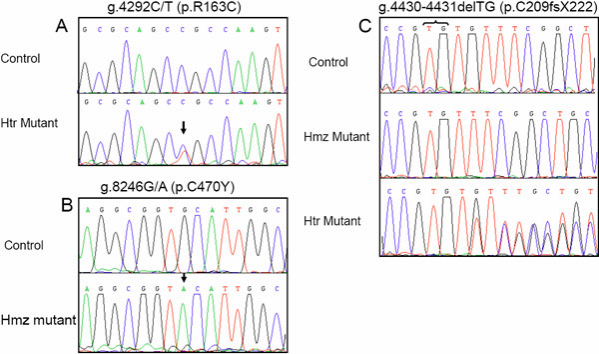
Detection of three novel *CYP1B1* mutations in Moroccan PCG families by direct DNA sequencing. Sequencing results of probands from families PCG-101 (**A**), PCG-100, and PCG-64 (**B**) and PCG-84 (**C**). Chromatogram of a heterozygous subject for g.4330–4331delG is shown in **C**. The names of the mutations and their corresponding amino-acid change are indicated above each chromatogram. Control sequences are shown for comparison purposes. Arrows indicate the changed nucleotides and curley bracket the deleted nucleotides in the g.4330–4331delTG allele. Because of the presence of a three GT repeat in this region, it is not possible to determine exactly which of the positions is deleted. Therefore, the position of this deletion was arbitrarily indicated. Htr: heterozygous mutation and Hmz: homozygous mutation.

Eight of these mutations have been previously described as disease-causing in different populations (p.G61E, p.E173K, p.V364M, p.R368H, p.R390S, p.R469W, g.4339delG, and g.7901–7913del13bp) [2,34,35]. An additional previously reported mutation p.E229K was detected in the mother of patient PCG-64-III-1 (Figure 1). To the best of our knowledge, the remaining mutations p.R163C (rs104894978), p.C470Y (rs104894979), and g.4330–4331delTG (rs104894980) were novel (Figure 2).

The detected mutations were present in the probands as follow: 34 (37.77%) were homozygous (24 for g.4399delG, five for p.G61E, one for p.R390S, one for p.C470Y, one for p.R469W, one for g.4430–4431delTG, and one for g.7901–7913del13bp). Seven mutations (7.77%) were compound heterozygous (two g.4339delG/ p.G61E, two g.4339delG/p.E173K, one g.4339delG/p.V364M, one g.4339delG/g.4430–4431delTG, and one g.4339delG/g.7901–7913del13bp). Two (2.22%) were heterozygous carrying only one mutated allele (p.R163C and p.R368H).

No other mutations were detected in the probands. Only 14 polymorphic variants were identified: g.3748G>A, g.3791C>T, g.3793T>C, g.3947C>G (p.R48G), g.3952C>A (p.S49S), g.4160G/T (p.A119G), g.4369C/A (p.G188G), g.4534G/C (p.V243V), g.7870C/A, g.7915T/C (p.L360L), g.8131C>G (p.L432V), g.8165C>G (p.A443G), g.8184T/C (p.D449D), and g.8195A>G (p.N453S). Two of them: g.3748G>A (ss244236418) and g.7870C/A (ss244236419) are reported herein for the first time. These intronic variations were found in both PCG chromosomes and control chromosomes. The frequency of g.3748G>A was 0.93% (1/108) in the sequenced PCG chromosomes and 1% (2/200) in control chromosomes. While, the frequency of g.7870C/A variation was 1.85% (2/108) in the sequenced chromosomes and 1.5% (3/200) in control chromosomes. This suggests that these substitutions are polymorphisms rather than causative-disease mutations.

### Novel *CYP1B1* mutations

The g.4330–4331delTG resulted in a frameshift after residue 208 leading to a premature stop codon in the helix-F, 13 amino-acids downstream from Val208 (p.C209fsX222). The g.4330–4331delTG was detected in two patients, one of them from a consanguineous family with two affected subjects and the other one was an isolated case of PCG. The first patient PCG-84-V.2 and his affected brother (PCG-84-V.3) were homozygous for g.4330–4331delTG and both parents were heterozygous for the same mutation. The patient PCG-64-III.1 was compound heterozygous for g.4339delG and g.4430–4431delTG. The father (PCG-64-II.3) and the paternal grandmother (PCG-64-I.2) were heterozygous for g.4339delG. The healthy mother (PCG-64-II.4) was heterozygous carrier of g.4330–4331delTG. Intriguingly, her second allele carried the p.E229K mutation.

The other novel missense mutations p.R163C and p.C470Y lie in the loop connecting C- and D-helices, and HBL (heme binding loop) region of CYP1B1, respectively. The p.R163C mutation was identified in a non consanguineous family (Figure 1 and Figure 2). The proband (PCG-101-III.3), the father (PCG-101-II.1) and the paternal grandmother (PCG-101-I.2) were heterozygous for the same mutation. One of the unaffected sisters (PCG-101-III.2) and the paternal grandfather (PCG-101-I.1) didn’t carry the mutation. The mother (PCG-101-II.2) was heterozygous for p.G61E. Surprisingly, the patient (PCG-101-III.3) didn’t carry this mutation, while his sister (PCG-101-III.1) was heterozygous. No mutation was found in the coding region of *MYOC* in the proband (PCG-101-III.3) except previously reported polymorphisms.

The p.C470Y mutation was homozygous in the proband PCG-100-II.3 belonging to a consanguineous and multiplex family. Unfortunately, the DNAs of the other family members were not available for further screening. None of the novel mutations was found in 100 unrelated control subjects.

### *CYP1B1* haplotype analysis

Single nucleotide polymorphisms (SNPs) (g.3947C/G, g.4160G/T, g.8131/G, and g.8195A/G) were analyzed in PCG patients and normal controls. As the first step of our screening strategy was based on the screening of g.4339delG and p.G61E by PCR-RFLP, only few probands (eight) carrying these mutations were sequenced and their haplotype constructed and only patients in whom a complete haplotype could be determined was included. Therefore, the haplotypes of a total of 20 PCG probands with *CYP1B1* mutations, 46 PCG probands without *CYP1B1* mutations, and 100 normal controls were analyzed.

C/G/G/A haplotype was the most common haplotype (75%, 15/20) among the patients who carried *CYP1B1* mutations (Table 3). Moreover, the C/G/G/A haplotype background was associated with seven of the 11 identified mutations (p.G61E, p.E173K, g.4339delG, g.7901–7913del13bp, p.V364M, and p.R469W). However, among the patients without mutations and normal controls, the most frequent haplotype was C/G/CG/A (41.3%, 19/46 and 40%, 40/100; respectively) (data not shown).

**Table 3:** *CYP1B1* single nucleotide polymorphisms and mutations detected in 20 probands with primary congenital glaucoma.

**Patient ID**	**Mutation**	**g.3947C>G (p.R48G)**	**g.4160G/T (p.A119G)**	**g.8131C>G (p.L432V)**	**g.8195A>G (p.N453S)**
PCG-3-III.3	g.4339delG/g.4339delG	C/C	G/G	G/G	A/A
PCG-4-IV.3	g.4339delG/g.4339delG	C/C	G/G	G/G	A/A
PCG-17-III.3	g.4339delG/g.4339delG	C/G	G/G	G/G	A/A
PCG-25-IV.3	g.4339delG/g.4339delG	C/G	G/G	G/G	A/A
PCG-1-II.5	p.E173K/g.4339delG	C/G	G/G	G/G	A/A
PCG-28-II.2	p.E173K/g.4339delG	C/C	G/G	G/G	A/A
PCG-64-III.1	g.4330–4431delTG/g.4339delG	C/C	G/G	G/G	A/A
PCG-84-V.2	g.4330–4431delTG/g.4330–4431delTG	C/C	G/G	G/G	A/A
PCG-95-III.2	g.4339delG/g.7901-7913del	C/C	G/G	G/G	A/A
PCG-11-II.1	g.4339delG/p.V364M	C/C	G/G	G/G	A/A
PCG-58-V.2	g.79017913del/g.7901-7913del	C/C	G/G	G/G	A/A
PCG-79-III.3	g.4339delG/p.G61E	C/G	G/G	G/G	A/A
PCG-47-II.3	p.G61E/p.G61E	C/G	G/G	G/G	A/A
PCG-89-V.3	p.G61E/p.G61E	C/G	G/G	G/G	A/A
PCG-97-II.3	p.G61E/p.G61E	C/G	G/G	G/G	A/A
PCG-14-II.2	p.R368H/w	C/C	G/G	C/G	A/G
PCG40-IV.6	p.R390S/p.R390S	C/G	T/T	G/G	A/A
PCG-75-IV.1	p.R469W/p. R469W	C/C	G/G	C/C	A/A
PCG-100-II.3	p.C470Y/p.C470Y	C/G	G/T	C/C	A/A
PCG-101-III.3	p.R163C/w	G/G	G/G	C/G	A/A

Only probands in whom a complete haplotype could be determined are included. w: normal allele.

### *MYOC* sequence analysis

Sequence analysis of *MYOC* revealed a heterozygous substitution (g.16 072C/A) in only one patient (PCG9-II.3, Figure 1), resulting in a missense mutation (p.T293K). This mutation has been previously reported in patients with POAG and ocular hypertension [26,36-38]. The p.T293K was not found in 98 control subjects. This is the first time that this mutation is reported in patient with PCG. No other disease-causing mutations were found. Only single nucleotide polymorphisms previously described were identified in exon 1 (g.349G/A, p.R76K; g.388C/T, p.G122G; g.445C/T, p.A141A) and exon 3 (g.16169 G/A, p.T325T; g.16235C/A, p.Y347Y).

## Discussion

This is the first report of the *CYP1B1* mutation spectrum in a large sample of Moroccan families. It is also the first time that screening of *MYOC* has been reported in Morocco. *CYP1B1* is considered as a major cause for PCG in different populations and mutations have been reported with variable frequency depending on ethnic and geographical differences [2,3].

### *CYP1B1* mutation frequency in primary congenital glaucoma in Morocco

In this study, 47.77% (43/90) of probands with PCG had *CYP1B1* mutations. This percentage is similar to the one reported in French (48%) [23], Brazilian (50%) [39], and Indian (40%) [40] populations. However, it is lower than the 70 to 100% percentage previously reported in the more homogeneous and inbred populations of Slovakia Roma [41], Saudi Arabia [5], Kuwait [42], and Iran [35]. Our mutation rate is also higher than the percentages reported in ethnically mixed populations such as Indonesian (22.2%) [43], Japanese (20%) [44], Australian (21.62%) [24], and Chinese (17.2%) [9]. It is also slightly higher than the corresponding figures of Spanish (34%) [25] and Moroccan PCG (34%) [28] populations. This difference is probably due to the patients’ geographical locations, as well as the sample composition (familial/sporadic, consanguinity rate, unilateral/bilateral) and size. Among the 43 mutated probands, consanguinity was found in 29 cases (67.44%), and 19 (44.18%) of them had a positive family history. Homozygosity of the mutant alleles was found in 79.06% (34/43), and compound heterozygosity in 16.27% (7/43) of the cases. Among the mutated patients two (4.65%) were heterozygous for only one mutation in *CYP1B1*. In each family, when relatives are available, the mutant alleles segregated with the disease phenotype in an autosomal recessive pattern, except in pedigree PCG-64 in which the healthy mother carried two potentially mutated alleles (Figure 1). This case is discussed below.

### *CYP1B1* mutations in primary congenital glaucoma in Morocco

Among the 12 detected mutations, seven missense mutations (p.G61E, p.E173K, p.E229K, p.R368H, p.V364M, p.R390S, and p.R469W), and two deletions (g.4339delG and g.7901–7913del13bp) were previously reported in patients from different populations [2]. To the best of our knowledge, three of them were novel (p.R163C, p.C470Y, and g.4330–4331delTG) and five (p.R368H, p.V364M, p.R390S, p.R469W, and g.7901–7913delG13bp) are identified herein for the first time in African population. The remaining mutations (p.G61E, p.E173K, and g.4339delG) have been previously reported in different populations [2].

It is noteworthy that four of these mutations (p.G61E, p.E229K, p.R368H, and g.7901–7913del13bp) were previously found in patients with POAG, Rieger’s syndrome or Peters anomaly, and therefore are associated with different glaucoma phenotypes [2]. Previous structural and/or functional studies showed that the previously described missense mutations alter at least one of the protein properties, demonstrating their pathogenic character [25,45-47].

CYP1B1 is a member of the cytochrome P450 superfamily which shares a highly conserved COOH-terminal core (CCS) involved in the heme-binding ability of the structure. Human CYP1B1 is a 543 amino-acid long protein which is made up of three regions: the 53 residue-long membrane-bound NH_2_-terminal region, a 10 residue-long proline rich region called “the hinge region,” and the 480 residue-long cytosolic globular domain which contains the CCS elements. This region includes four helix- bundles (helices D, I, L, and the antiparallel helix E, helices J and K, beta-sheets 1 and 2, the heme-binding region, and the meander just NH_2_-terminal to the heme-binding region [48,49]. The heme-binding region which harbors the invariant cysteine of all known P450 proteins (i.e., Cys470 of CYP1B1) is essential for the normal function of every P450 molecule [50]. All the mutations reported herein, except p.G61E are located in the cytosolic domain.

#### Novel mutations

The novel mutations we reported were expected to be pathogenic based on the following criteria: the causing frame shift and creating of a stop codon (g.4330–4331delTG), the nature of the amino-acid change it caused, the degree of conservation, the absence of mutations in controls, and Polyphen, PolypPhen-2, and SIFT prediction (p.R163C and p.C470Y). These algorithms were recently used to predict whether a missense mutation is likely to have or not a deleterious effect [51-54].

The 4330–4331delTG mutation was found in two patients carrying null alleles. It was homozygous in proband PCG-84-V.2, and compound heterozygous (g.4339delG/g.4330–4331delTG) in proband PCG-64-III.1. It was predicted to truncate the CYP1B1 protein after the amino-acid 221 of F-helix. The resulting protein therefore lacked a large part of the important cytosolic domains. Because the COOH-terminal half of the CYP1B1 protein is expected to be involved in heme-binding and proper folding of the molecule [55], these null allele eliminating the essential part of the P450 protein obviously affect the structure and damage the function of CYP1B1.

**Figure 3:**
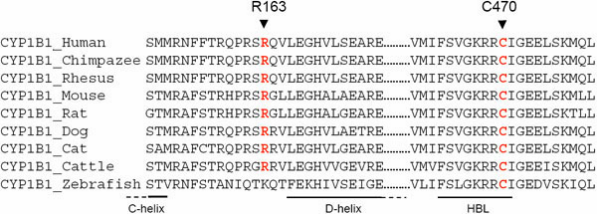
Multiple amino-acid sequence alignment of CYP1B1 from different species. Sequence alignment was generated by ClustalW. The positions of mutated amino-acids newly reported in this study are indicated by arrows and red letters. The COOH-terminal amino-acids of C-helix, NH_2_-terminal amino-acids of D-helix and heme binding loop (HBL) are indicated below the sequence alignment, by a line.

The p.R163C mutation site is located in the COOH-terminal of the cytosolic loop connecting C- and D-helices. It occurred at conserved position in chimpanzee, rhesus monkey, cattle, mouse, rat, cat, and dog CYP1B1, although not in zebrafish. Furthermore, a cysteine is never found in any CYP1B1, whatever the species analyzed at this position (Figure 3). The p.R163C was not found in 200 control chromosomes. Substitution of arginine to cysteine leads to a change from a positively charged residue to a hydrophobic and uncharged amino-acid. This may affect the charge distribution, and may destabilize ionic interactions, and thus could cause a conformational change. The SIFT score of p.R163C was 0.01 and is predicted to affect the protein function. The PolyPhen PSIC score of this mutation was 1.54 indicating that this change is possibly damaging. Polyphen-2 program indicated that this mutation is probably damaging. These data suggested that p.R163C is a pathogenic mutation rather than a benign polymorphism.

The other novel mutation, p.C470Y, was homozygous in patient PCG-100-II.3 from a consanguineous family. This change substituted a hydrophobic residue to a hydrophilic amino-acid. At this position, the cysteine is absolutely invariant (Figure 3) among the P450 family and is a part of the “signature sequence” (NH_2_-FXXGXXXCXG-COOH) that is present in all heme binding cytochromes at the COOH-terminus of the protein (serving as the axial ligand of the heme iron). The cysteine in this sequence acts as the fifth ligand to the heme [27]. This change is expected to interfere with the ability of mutated protein to perform normal physiologic functions and probably affects severely the normal metabolism of the other molecules that require CYP1B1 participation. The SIFT score of p.C470Y was 0.00 and is predicted to be deleterious for the protein function. The PSIC score of this mutation was 4.173 indicating that this mutation is probably damaging. PolyPhen-2 software confirmed this result. These data strongly suggested that p.C470Y which affect a critical amino-acid of CYP1B1, is a pathogenic mutation.

Few reported studies showed that genotype/phenotype correlation varies, depending on the type of mutation [56,57]. Our data showed that the novel mutations are associated with moderate (p.R163C) to severe (p.4330–4331delTG and p.C470Y) phenotypes, based on the severity index proposed by Panicker et al. [56]. This is not surprising since the patients PCG-64-III.1 and PCG-84-V.2, carried deletions which are suggested to create a null allele and p.C470Y affected a crucial amino-acid of CYP1B1 and present at the homozygous state.

#### Most frequent mutations

In our study, g.4339delG was found to be the most frequent change, followed by p.G61E. These two mutations together were present in 37.21% (30.55 and 6.66%, respectively) of the Moroccan studied alleles. The g.4339delG mutation was initially described in Moroccan patients with a comparable rate (25.80% of alleles) [28]. It is also the most frequent *CYP1B1* mutation among patients in Brazil [39] (21% of chromosomes) with a lower percentage than in Moroccan population.

This relatively high frequency of g.4339delG, in Morocco, is likely due to a founder effect as it has been previously suggested [28], as well as its ancient appearance in Moroccan population [28]. In our study, g.4339delG was found at the homozygous state in 24 of the 90 studied probands (26.66%) and present in all of compound heterozygous. This is consistent with the relatively high frequency of consanguinity (estimated at approximately 30%) and its rate in Morocco. Although the p.E173K, p.V364M, and g.7901–7913del13bp mutations have been previously described, their association with g.4339delG is novel.

#### Rare mutations

All the remaining reported mutations were infrequent in our PCG patient’s sample, each detected in less than 2% (0.55%–1.66%) of the tested chromosomes. Similarly to our study, the p.E173K, p.R390S, and g.7901–7913del13bp mutations have been reported at a very low frequency [2,34,35]. So far, a few PCG families were found to have these mutations. However, p.R368H, p.V364M and p.R469W have been reported to be among the most common mutations in different populations [5,35,40,43,58,59].

### *CYP1B1* haplotype background

Most of the mutations identified herein shared a common haplotype C/G/G/A (Table 3). This haplotype has been previously reported in PCG patients from Saudi Arabia, Morocco, Turkey, and India indicating that founder effects must have occurred for most *CYP1B1* mutations [5,28,60]. Particularly, in Moroccan population, a founder effect has been suggested for g.4339delG [28]. Our data agree with the founder effect hypothesis for p.G61E, g.4339delG, p.E173K, g.4330–4331delTG, and g.7901–7913del13bp. However, an extended haplotype analysis is required to confirm this hypothesis.

### Variable expression and incomplete penetrance of the PCG phenotype

It is noteworthy that the p.R390S mutation was associated with PCG and POAG, in a consanguineous family (PCG-40). The proband (PCG-40-IV.6) and his paternal uncle (PCG-40-III.1) were homozygous for this mutation. The proband (PCG-40-IV.6) had a neonatal and aggressive form of PCG with severe corneal changes, uncontrollable IOP higher than 50 mmHg leading to the evisceration of the right eye at the age of 10 years. Despite early and multiple surgical procedures, the visual outcome was poor in this patient.

The residue R390 is located in the conserved alpha K-helix and is a part of the consensus GluXXArg, which is absolutely conserved among all members of the cytochrome P450 family [48]. Structural data analysis suggested that the p.R390S, along with p.R390H and p.R390C, could affect the stability of the CYP1B1 protein [5,47]. Therefore, this homozygous mutation could explain the severe phenotype in patient PCG-40-IV.6. Intriguingly, the uncle (PCG-40-III.1) was diagnosed with POAG at the age of 45 years, showing the association of p.R390S with different glaucoma phenotypes. This suggests that additional factors are necessary for the development of the glaucomatous process [26]. The molecular association between PCG and JOAG or POAG has been documented in few cases with *CYP1B1* mutations [13,24,25], but this is the first time that p.R390S has exhibited variable expressivity of glaucoma.

It is interesting to note that p.E229K has been found in a healthy 29-year-old mother (PCG-64-II.4) who carried g.4430–4431delTG in her second *CYP1B1* allele. She was recently re-examined and had no symptoms of glaucoma. This mutation has been previously described in PCG patients and also in healthy carriers [25,56,61]. The association of p.E229K with a null allele raised the question of its potential pathogenicity. A similar situation has been previously reported (p.E229K/c.1064–1076del) [61]. Recently, functional studies classified the p.E229K variant as a hypomorphic allele with decreased function, and suggested that it could function as a risk allele, which can lead to the development of glaucoma in the presence of modifiers or environmental influence. It has also been proposed that reduced penetrance could be due to a dominant suppressor of the PCG phenotype, which is not linked genetically to *CYP1B1*, and/or the inducibility of *CYP1B1* by environmental lipophilic agents to which individuals may have been exposed [45]. Therefore, the mother (PCG-64-II.4) is probably a case of incomplete penetrance or delayed onset of glaucoma.

### *MYOC* mutation associated with PCG

Previous studies reported that mutations in *MYOC* or both of *CYP1B1* and *MYOC* have been detected in a small proportion of PCG cases [7-9,20]. In our cohort, *MYOC* was involved in only one patient (PCG-9-II.3) who was heterozygous for the p.T293K mutation, suggesting a minor role of *MYOC* in the Moroccan population. This mutation has been initially reported in patients with POAG or ocular hypertension [26,36-38], and this is the first report of the association of p.T293K with PCG. The proband PCG-9-II.3 carrying this mutation had advanced glaucoma at presentation and intervened belatedly. The outcome was bad in his case.

In our study three patients were heterozygous for only one mutation in *CYP1B1* or *MYOC*, and 46 (51.11%) didn’t carry any mutation. These results suggest the possibility of other candidate genes, or loci, not yet identified, that may be involved in anterior chamber development as it has been suggested by previous studies [10-12]. Given that only the coding region of *CYP1B1* and *MYOC* was sequenced, it is possible that a promoter or another non-coding variant may be present in heterozygous patients and among those who had no mutations.

In summary, we identified three *CYP1B1* novel mutations in addition to the eight previously reported, and one *MYOC* mutation previously associated with POAG. Nearly 48% of the patients carried *CYP1B1* mutations; most of them (46%) had two mutant alleles, with predominance of the g.4339delG mutation. Thus, in Morocco, mutations of *CYP1B1* are a major cause for PCG, while *MYOC* gene plays a minor role. Our results will be useful for genetic testing and genetic counselling, especially for the g.4339delG mutation in Moroccan patients. A better understanding of the genetics of PCG will contribute to early diagnosis and prevention of this severe blindness condition. Finally, despite the significant role of *CYP1B1* in Moroccan PCG, it is clearly evident that other genetic and/or environmental factors are still to be identified and that a concerted effort to identify the causative genes would be useful.
